# Targeting tumor-associated macrophages in hepatocellular carcinoma: biology, strategy, and immunotherapy

**DOI:** 10.1038/s41420-023-01356-7

**Published:** 2023-02-15

**Authors:** Hongyu Zheng, Xueqiang Peng, Shuo Yang, Xinyu Li, Mingyao Huang, Shibo Wei, Sheng Zhang, Guangpeng He, Jiaxing Liu, Qing Fan, Liang Yang, Hangyu Li

**Affiliations:** Department of General Surgery, The Fourth Affiliated Hospital, China Medical University, Shenyang, 110032 China

**Keywords:** Immunology, Diseases

## Abstract

Hepatocellular carcinoma (HCC), one of the most malignant tumors, is characterized by its stubborn immunosuppressive microenvironment. As one of the main members of the tumor microenvironment (TME) of HCC, tumor-associated macrophages (TAMs) play a critical role in its occurrence and development, including stimulating angiogenesis, enhancing immunosuppression, and promoting the drug resistance and cancer metastasis. This review describes the origin as well as phenotypic heterogeneity of TAMs and their potential effects on the occurrence and development of HCC and also discusses about various adjuvant therapy based strategies that can be used for targeting TAMs. In addition, we have highlighted different treatment modalities for TAMs based on immunotherapy, including small molecular inhibitors, immune checkpoint inhibitors, antibodies, tumor vaccines, adoptive cellular immunotherapy, and nanocarriers for drug delivery, to explore novel combination therapies and provide feasible therapeutic options for clinically improving the prognosis and quality of life of HCC patients.

## Facts

- Tumor-associated macrophages are one of the most abundant immune cells present in the tumor microenvironment of hepatocellular carcinoma, and have been implicated in both occurrence and development of hepatocellular carcinoma.
- Targeting tumor-associated macrophages can serve as an important strategy for the clinical management of hepatocellular carcinoma in the future, and macrophage-based immunotherapy can gradually be incorporated into the clinical regimen.
- Growing evidence suggest favorable prognosis and promising trends of tumor-associated macrophages as therapeutic targets.

## Open questions

- Where do the macrophages originate from in liver cancer?
- What are the therapeutic strategies targeting tumor-associated macrophages?
- What are the immunotherapeutic options based on macrophages?

## Introduction

According to the global cancer data for 2020 recently released by the WHO, liver cancer ranks seventh in morbidity and third in mortality among different cancers [1]. Hepatocellular carcinoma (HCC), the most common type of liver cancer, accounting for ~80% of primary liver cancers, and remains one of the most common as well as leading causes of mortality worldwide [2, 3]. The intractable tumor microenvironment (TME) plays an vital role in the development and progression of HCC and constitutes one of the three key unsolved issues (cancer recurrence, fatal metastasis, and the refractory tumor microenvironment) that can obstruct effective cancer management in the clinical practice [4, 5]. The tumor microenvironment (TME) characterizes all the noncancerous components near the tumor cells, including fibroblasts, myelogenic suppressor cells (MDSCs), macrophages, lymphocytes, the extracellular matrix (ECM), and interwoven blood vessels generated by endothelial cells and pericytes, thereby creating a protective niche in which the tumor cells are immune to routine intervention, leading to the treatment failure [6]. Although the ‘seed and soil’ theory was initially proposed in 1889, only in recent years increasing attention have been paid to the role of the TME in promoting tumor development [6]. Over the past decade, cognitive expansion about how the TME can potentially interact with the tumor cells has led to exploration of a new tumor treatment model: targeting the tumor stromal cells. However, the design of novel therapies that can target the tumor stroma of HCC relies primarily on exhaustive comprehension of the mutual effects between the TME and HCC cells.

The TME is a complex multicellular system characterized by tumor cell-stromal cell-extracellular matrix interactions [6]. Tumor-associated macrophages (TAMs), the number of which in the TME accounts for 20% to 40% among all HCC patrolling and infiltrating lymphocytes and even more in some rare HCC subtypes, have been reported to be enriched in the TME of HCC and can coordinate with the tumor-associated inflammation [6–13]. In addition, tumor-associated macrophages (TAMs), which infiltrate most of the solid tumors in abundance, can contribute to tumor progression by providing a major barrier against antitumor immunity and by stimulating proliferation, angiogenesis, and metastasis [14]. Moreover, in terms of mechanism, TAMs can establish and reshape the structure of the extracellular matrix such that the tumor cells are able to invade the TME and interact with other tumor or stromal cells by secreting various cytokines such as interleukin, interferon, tumor necrosis factor superfamily, colony-stimulating factors, chemokines, and growth factors [15, 16]. It has been established that TAMs cannot not be regarded as a homogenous cell population, and these cells can either promote or inhibit tumors in different systems or even exhibit both the functions [16, 17]. In-depth molecular studies of TAMs in the human malignant tumors have expanded our understanding of their source complexity, phenotypic heterogeneity, and functional diversity. In principle, identification of the specific oncogenic TAM subtypes can pave the way for the development of novel as well as optimal TAM-targeted anticancer immunotherapy.

In this review, we have described the origin and heterogeneity of TAMs and discussed the role of TAMs in regulating the initiation, progression, metastasis, and drug resistance of HCC. We have also described in detail existing therapeutic strategies and feasible immunotherapy options for targeting TAMs in HCC to accelerate the leap from laboratory research to rapid clinical application.

## Biology of TAMs in HCC

### Origin of TAMs

**Fig. 1:**
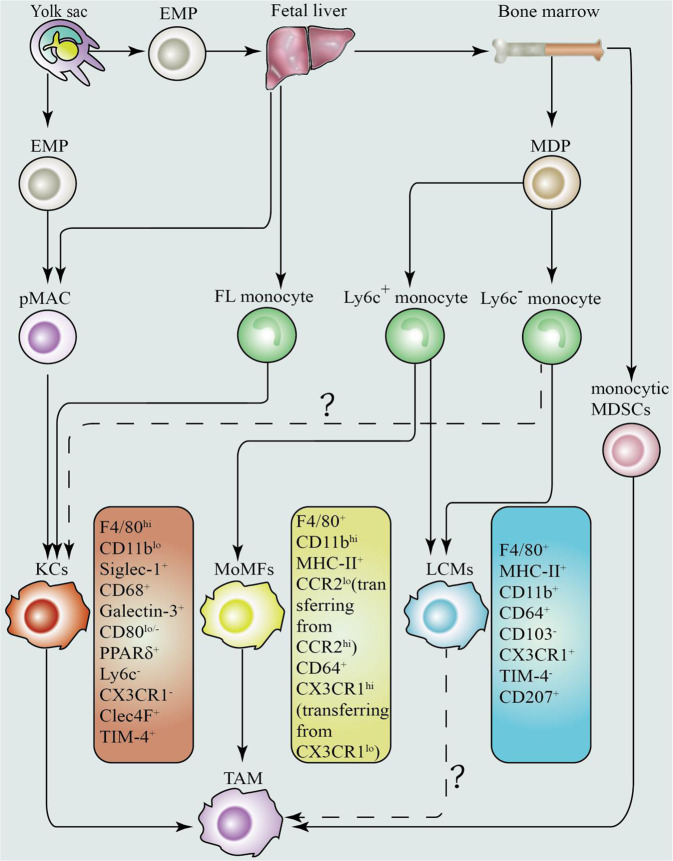
There are three major sources of TAMs in HCC: yolk sac, fetal liver, and bone marrow, as well as three direct sources: blood/bone marrow-derived monocytes, KCs, and MDSCs. YFP^+^ AA4.1^+^ Kit^+^ CD45^lo^ EMPs (erythro-myeloid progenitor), which appeared initially in the yolk sac of a mouse embryo at E(day)8.5, have been identified as macrophage progenitor cells [135]. EMPs, which can exist as Kit^+^ progenitor cells can effectively migrate and proliferate in the fetal liver until E16.5 and produce the fetal liver monocytes (appearing at E12.5) until the late stage of fetal development [135]. Moreover, EMPs have been found to rapidly differentiate into premacrophages (pMacs), which can simultaneously colonize the whole embryo from E9.5 in a CX3CR1-dependent manner and differentiated into macrophages [136]. It has been reported that starting from E10.5, almost immediately after embryonic tissue colonization, YFP^+^F4/80^bright^ fetal macrophages can appear in the fetal liver [135, 136]. Thereafter, around E14.5, fetal liver monocytes can replace early F4/80^hi^ macrophages and constitute most of the liver-resident macrophages in the adult liver, that is, Kupffer cells (KCs) [135, 137]. During the postnatal bone formation, the hematopoietic function of the fetal liver decreases significantly and is replaced by BM [135, 138, 139]. BM-derived mononuclear phagocyte precursor MDPs (macrophage/dendritic cell progenitor cells) which can produce inflammatory monocytes (Ly6c^+^), giving rise to the patrol monocytes (Ly6c^−^) [138, 139]. Interestingly, both inflammatory monocytes (Ly6c^+^) and patrol monocytes (Ly6c^-^) can be recruited into the liver capsule and then differentiate into CD207-EGFP^hi^ F4/80^+^ liver capsular macrophages (LCMs) [140, 141]. Inflammatory monocytes (Ly6c^+^) enter the liver and participate in the formation of the resident macrophages in a very small proportion of the tissues, namely, monocyte-derived liver macrophages (MoMFs) [31, 135, 139]. Both KCs and blood-recruited MoMFs have been found to be involved in the formation of the tumor-associated macrophages, but it is not clear whether LCMs are also involved [18, 19]. LCMs have been also rarely considered as a predecessor of TAMs because of their special anatomical location [140]. In addition, MDSCs can differentiate into immunosuppressive TAMs in the microenvironment of HCC in the presence of HIF-1α or hypoxia [142, 143]. Collectively, the precise origins of TAMs have not been fully elucidated.

The source of TAMs is heterogeneous (Fig. 1). Tissue-resident macrophages (so-called Kupffer cells) in the liver and blood-recruited monocyte-derived macrophages have been reported to be involved in the formation of the TME [18, 19]. However, it has been hypothesized that TAMs are derived primarily from the circulating blood monocytes [20]. In the mice, TAMs are mainly derived from bone marrow monocytes (TAMs in human HCC arise from CCR2^+^ monocytes) that can recruited by inflammatory signals released by cancer cells in both the primary and metastatic tumors, and they can differentiate into TAMs under the action of chemokines and growth factors produced by the stromal cells and tumor cells, thus promoting tumor progression [20–23]. For example, an increase in TAMs induced by lysyl oxidase-like 4 (LOXL4: a copper-dependent monoamine oxidase in the extracellular matrix) in mice has been found mainly due to monocyte infiltration; LOXL4 can inhibit the proliferation of resident macrophages in the liver and nearly deplete the resident macrophages during the formation of HCC [24]. In addition, recent studies have indicated that tumor-dependent recruitment of monocyte-derived macrophages occurs in chronically damaged liver tissues compared with the tumors growing in healthy livers [25]. Furthermore, self-replication can serve as an important mechanism for facilitating accumulation of tumor-infiltrating macrophages in HCC tissues [26]. It is worth noting that evidence also suggest that macrophages in the liver tissues can be established by progenitor cells from the yolk sac and fetal liver and maintained by the self-proliferation and monocyte input [27–29]. For instance, Yu-Chen Ye et al. found that the dominating TAMs in orthotopic HCC in conditional disruption of the recombination signal binding protein Jκ (RBPj cKO) mice manifested properties of Kupffer cells (KCs), thus suggesting that TAMs (KC-like TAMs, F4/80^+^CD11b^lo^Ly6c^lo/−^ TAMs) in HCC in situ were more likely to originate from embryonic hematopoiesis–generated KCs and bone marrow (BM) monocyte-derived KCs, which are considered as the self-renewing tissue-resident macrophages [30–32]. However, it is not clear whether these KC-like TAMs can originate from true KCs, mononuclear cells derived from bone marrow or extramedullary origin, or even monocyte-derived TAMs (moTAMs). KC-like TAMs differentiate from true KCs or bone marrow-derived or extramedullary monocytes. However, KCs might account for only a small part of the total TAM pool of HCC [30]. These observations reinforce that the definition of TAMs in HCC should not only be used to identify bone marrow-derived macrophages that can infiltrate the tumors but should also be extended to all the macrophages that play a vital role in the TME.

TAMs can originate from the different sources and play diverse protumoral or sometimes antitumoral roles. Interestingly, each subpopulation has a characteristic transcriptional landscape and marker profile based on the type, stage, and immune composition of the tumors they can infiltrate [33]. Lineage tracing [34] is often used to track the origin of the tumor cells, and this technique has also been employed to identify the cellular origin of TAMs. Thus, a fascinating suggestion that monitoring the fate of TAMs during the evolution of HCC can lead to a more thorough understanding of the complex nature of the TME. Single-cell RNA sequencing technology [35, 36] can be used to reveal the TAM type at the single-cell level, and spatial transcriptomics [37] can aid to visualize the different proteins or expressed genes, the combination of which can provide a more theoretical basis to support the application for targeted therapy.

### Phenotypic heterogeneity of TAMs

Macrophages have long been considered to have two different activation states: Macrophages with inflammatory functions are called M1 macrophages, and macrophages with anti-inflammatory functions are termed as M2 macrophages [38, 39]. Most TAMs exhibit M2 polarization (cell morphology tends to be more fusiform [40, 41]) and can promote progression of HCC by secreting the various protumor and angiogenic factors and inhibiting activation of tumor-infiltrating T cells. In addition, along with the transition from the M1 to the M2 state, macrophages acquire features that can effectively promote tumor invasion, metastasis, and immunosuppression with upregulated expression of genes such as *MMP14* (matrix metalloproteinase 14), *VEGFA* (vascular endothelial growth factor A), and *MRC1/CD206* (mannose receptor) [13]. However, macrophages can gradually obtain the characteristics of the M2 phenotype, but the M1 signature is not obviously reduced [13]. It has been reported that M2-like macrophages can maintain some antitumor properties, which supports the view that macrophage activation in the TME of HCC does not follow the classical polarization pattern. In the similar fashion, a study suggested that the two macrophage subsets (Mø_c2 and Mø_c3) are maintained in the transition state mentioned which can facilitate the transition from M1 polarization to M2 polarization of macrophages is gradual in the progression of HCC. The understanding of TAMs could be potentially extended to all macrophage subsets in the TME because each kind of macrophage inevitably can effectively exchange materials and information with the tumor cells, which is closely related to tumor progression, either promoting or inhibiting tumor progression.

The phenotypic heterogeneity of TAMs is primarily manifested by a wide range of biological markers with selective expression patterns in the context of a specific TME [42]. In mice, TAMs are identified in the tumors as F4/80+ and CD11b^+^. In humans, TAMs are identified as CD68^+^ by immunohistochemistry and CD14^+^ by flow cytometry. There are several other markers used to define HCC TAMs [43]. M1-phenotype TAMs polarized from the peripheral blood mononuclear cells (PBMCs) are marked by relatively higher expression of IL-1β, TNF-α, IL-6, IL-12, HLA-DR(HLA-DRα), CCR7, Type I IFNγ, CXCL1–3, CXCL-5, CXCL8–10, CCL10, inducible nitric oxide synthase (iNOS or NOS2), MHC II, CD11c, CD80, CD86 and CD16/32 in HCC [44–55]. Conversely, M2-phenotype TAMs polarized from PBMCs are marked by higher expression of CD209 (DC-SIGN), CD206 (MRC1), CD204 (MSR1/SR-A), CD163, CD115, IL4, IL10, Fizz1, p-STAT3 and Arg1 [41, 56–75] (Table 1). Moreover, similar to cancer-associated fibroblasts (CAFs) and T lymphocytes, TAMs comprise a group of distinct cell subsets that can respond differently to different interstitial stimuli, exhibit unique secretory phenotypes, and play specific biological roles in the TME. Thus, mastering reliable and specific cell surface markers is key to distinguishing TAM subsets. In addition, contradictory results obtained suggest that the pleiotropic function of TAMs can be attributed to the high heterogeneity of TAMs, and hence it is necessary to comprehensively describe the cell origin, surface markers, activation stages, and spatial distributions of TAMs to distinguish which TAM cell types are being studied in each experiment.

**Table 1:** Cytokines secreted by TAMs and their functions.

Classification	Family	Cytokines	Functions	References
M1	Interleukin, IL	IL-6	IL-6 can upregulate the expression of LIN28 in HCC progenitor cells (HcPCs), so that HcPCs can obtain autocrine IL-6 signals that stimulate its growth and malignant progression in vivo.	[44, 45]
		IL-1	IL-1 promotes recruitment of myeloid cells in tumors and their tumor-promoting function;IL-1 induces in endothelial cells and surrounding stromal cells the production of proangiogenic cytokines such as IL-8.	[46, 47]
		IL-12	IL-12 primes naive CD4 + and CD8 + T cells into T helper and cytotoxic cells.	[44, 48]
	Interferon, IFN	IFN-γ	TLR (Toll-like receptor) response induced by IFN- γ greatly enhanced the expression of inflammatory mediators and immune effectors induced by TLR, IFN- γ could exert its anti-tumor function by inducing apoptosis, and IFN- γ could play its tumor-promoting effect by promoting the expression of PD-L1 in tumor cells.	[49–51]
	Tumor necrosis factor, TNF	TNF-α	Synergistic inhibition of tumor growth by TNF- α and IFN- γ.	[44]
	Chemokine family	CXCL9	Expression of the chemokine CXCL9 by TAMs regulates the recruitment and positioning of CXCR3-expressing stem-like CD8 T (Tstem) cells that underlie clinical responses to anti-PD(L)-1 treatment.	[52–55]
		CXCL10	CXCL9 and CXCL10 co-localize with LAG3 + T cells expressing CCL4 or CXCL13 and contribute to the generation of a “hot” tumor microenvironment.	[52–54]
M2	Tnterleukin, IL	IL-10	IL-10 inhibits Th1 and Th2 CD4 + T cell helper functions, IL-10 expressing. anti-inflammatory macrophages are responsible for induction of iTreg cells.	[64–66]
		IL-6	IL-6 induces signal transduction and JAK/STAT3, MAPK/ERK or PI3K/AKT activation in tumor, leading to drug resistance.	[67]
	Transforming growth factor-β, TGF-β	TGF-β	TGF-β inhibits cytotoxic T lymphocyte, Th1 and Th2 CD4 + T cells; TGF-β induces LOX in CAFs to remodel ECM	[66, 68, 69]
	Chemokine family	CCL2/MCP-1	CCL2 induces the recruitment of circulating CCR2 + Ly6C+ monocytes into the liver.	[70]
		CCL3	CCL3 activates the Akt signaling pathway by binding to CCR5, CCL3-CCR5 axis facilitated COAD cell migration and invasiveness by activating the Akt signaling.	[71, 72]
		CCL17	CCL17 promotes pituitary adenoma invasion via the CCL17/CCR4/mTORC1 axis.	[73]
		CCL22	CCL22 promotes tumor immunosuppressive microenvironment and drug resistance.	[74, 75]

### Etiological background of TAM in HCC

The etiological background of TAM in HCC is extremely complicated. It has been reported that the viral infection, long-term drinking and fat accumulation can cause liver damage, liver injury promotes the recruitment of Ly6C^hi^ macrophages from the bone marrow, thus substantially increasing the number macrophages to the already-large number of liver-resident macrophages [76]. Moreover, liver-resident macrophages can act as promoters of inflammation and fibrosis in diseases such as viral hepatitis, alcoholic liver disease and non-alcoholic steatohepatitis. Interestingly, CCL2 can induce the recruitment of circulating CCR2^+^Ly6C^+^ monocytes into the liver [70]. Recruited monocytes are more pro-inflammatory than the resident Kupffer cells and can produce the various pro-inflammatory cytokines, such as TNFα and IL-1β that contribute to the development of NASH and fibrosis [70]. In the case of long-term inflammatory stimulation and fibrosis progression, HCC gradually formed. The existence of tumor not only can destroy the original spatial structure of the tissue to some extent, but also can damage the original niche of macrophages [77]. Macrophages, as immune cells, are originally involved in the regulation of immunological behavior against tumors, but after being modulated by the tumor cells, macrophages can be transformed into TAMs to promote the occurrence and development of tumors.

## Strategies for targeting TAMs

Most of the available treatment options are only effective against small-sized HCC (2–3 cm in diameter); very few systemic chemotherapies have been shown to be consistently efficacious in treating HCC [78, 79]. Current modalities for HCC treatment include transarterial chemoembolization (TACE), surgical resection, radiotherapy, chemotherapy, local radiofrequency ablation, and systemic targeted therapy [80, 81]. Tumor resection, liver transplantation, and local radiofrequency ablation are the most effective treatments for the management of early HCC, but they are not suitable for most patients with HCC, many of whom are already at an advanced stage at the time of diagnosis [81]. Although sorafenib, lenvatinib, which is noninferior to sorafenib, and regorafenib can increase survival and are used as standard treatments in advanced HCC [81], their effect is still not very effective. Hopefully, targeting TAMs in HCC as a feasible target for adjuvant therapy of HCC might lead to a better prognosis for patients with HCC [30, 41, 56, 59, 63, 75, 82–85](Table 2). What’s more, a better understanding of the recruitment and functional tilt of TAMs can provide a sound basis for the development of macrophage-centered therapeutic strategies. The negative prognostic significance of macrophage infiltration has been evaluated by traditional immunohistochemistry, molecular markers, or single cell analysis, which has promoted the clinical evaluation of macrophage targeted therapy strategy.

**Table 2:** Potential targets and therapeutic strategies for tumor-associated macrophages.

Strategies	Change of TAMs	Function method	Regulation pathway	Model of HCC	Consequence	References
Reducing infiltration		Ablation or pharmaceutical inhibition of CCR2	Cut CCL2-CCR2 axis	Hepa1-6	Inhibits the recruitment of mononuclear cells from bone marrow and reduces the generation of M2 TAM.	[30]
		Knock out osteopontin (OPN) gene	Downregulate the expression of osteopontin (OPN) or knock out the gene of osteopontin (OPN)	Hep3B and HepG2, Hepa1-6 and H22	Knockout of OPN gene significantly decreased the number of TAMs M2 macrophage markers and PD-L1 expression in tumor tissues of mice and combined with anti-PD-L1 and CSF1R inhibition induced effective anti-tumor activity.	[96]
		Ectopic expression of miR-26a	MiR-26a inhibits the expression of M-CSF by regulating the PI3K/Akt pathway	HepG2, HCCLM3	Ectopic expression of miR-26a in HCC cells inhibits M-CSF expression, macrophage infiltration in tumor and tumor growth.	[84]
		Adiponectin (a fat-derived hormone)	Downregulate ROCK/IP10/ angiopoietin-1/MMP9/VEGF cell signal in tumor tissue	MHCC97L、Huh7、Hep3B and PLC, orthotopic mouse model(orthotopic mouse model)	Adiponectin treatment inhibited hepatic stellate cell activation and macrophage infiltration, reduced microvessel density, significantly inhibited tumor growth and reduced the incidence of lung metastasis in liver tumors.	[85]
		Cerium oxide nanoparticles (CeO2 NPs)	Downregulate M1 gene involved in pro-inflammatory function	HepG2, Wistar rats	CeO2 NPs mainly accumulates in the liver, reduces macrophage infiltration and inflammatory gene expression, increases the apoptotic activity of liver tumor cells, and weakens tumor cell proliferation.	[86]
Inducing polarization	M2 to M1	PLX3397	Competition inhibits CSF-1R	MHCC97-H, MHCC97-L, HCCLM3, Hepa1-6, HepG2	PLX3397 treatment increased the number of CD8^+^ cells and the proportion of antigen-presenting macrophages, and inhibited tumor growth in both HCC xenograft models.	[41]
		MiR-98 mimic	Downregulate IL-10 expression via binding to the 3′UTR of IL-10	HepG2 and SMMC7721	MiR-98 upregulation helps to polarize M2TAMS to M1TAMS and inhibit TAM mediated HCC invasion (reduced invasion potential), migration, and epithelial-mesenchymal transformation	[83, 91]
		Decitabine; Etomoxi; GW9662	Hypomethylation of RIPK3 by inhibits FAO;caspase-1–dependent IL1β activation	Murine H22	Decitabine downregulated FAO by regulating PIPK3-ROS-caspase1-PPAR pathway, reversed the polarization of TAMS and inhibited the occurrence and development of HCC.	[56]
		Upregulate IL-37 expression	Down-regulate IL-6/STAT3 signaling	HepG2 and Huh-7	IL-37 inhibits the M2 polarization of TAM and thus inhibits the proliferation, invasion, and metastasis of HCC cells. In vivo, the upregulated expression of IL-37 in TAM under HCC conditions retarded tumor growth and progression.	[94]
		Baicalin	Baicalein downregulated TRAF2 in an autophagy-dependent pathway and promoted the continuous activation of the IKKα and RelB/p52 pathways.	MHCC97L; Murine orthotopic HCC model(BALB/cAnN-nu athymic mice)	Baicalin treatment for 5 weeks could completely inhibit the growth of HCC in situ in mice.	[87]
		Dual anti-PD-1/VEGFR-2 therapy	Regulates certain pathways in mononuclear macrophages and lymphocytes	HCA-1 in C3H mice and RIL-175 in C57Bl/6 mice	Normalized vessels and blockade of PD-1/PD-L1 axis lead to a reduction in Tregs and CCR2 + monocytes, shift from M2- to M1-type in tumor-associated macrophages, and promotion of cytotoxic T lymphocyte (CTL) infiltration and activation; the TME is reprogrammed to an anti-tumor state.	[97]
		MnO2-containing NPs(contains sorafenib and a unique MnO2 core)	Ameliorates the immunosuppressive TME by reducing the hypoxia- induced tumor infiltration of TAMs	The murine HCC cell line HCA-1 and the human HCC cell line JHH-7, Hep3B	By reducing hypoxia-induced M2 TAMs hepatocellular carcinoma infiltration, promoting macrophage polarization to M1 TAMs, increasing the number of CD8 + cytotoxic T cells in hepatocellular carcinoma, and reprogramming immunosuppressive TME, MnO2 nanoparticles (NPs) enhances the therapeutic strategy for PD-1/PD-L1 immune checkpoints and the effect of whole-cell tumor vaccine immunotherapy	[82]
		BisCCL2/5i mRNA-LNPs; trimeric PD-1 ligand inhibitor (PD-Li)	Blocking the expression of CCL2 and CCL5; Blocking PD-1/PD-L1.	Hepa1-6, orthotopic HCC-tumor-bearing mice	The combination of BisCCL2/5i mRNA-LNPs and trimeric PD-1 ligand inhibitor regulated the polarization of M2 TAM to M1, increased the proportion of CD8 + T cells and decreased the proportion of Tregs, thus alleviated immunosuppression and enhanced tumor-killing effect.	[92]
	M0 to M1	Upregulate RIG-I expression	Upregulate RIG-I/MAVS/TRAF2/NF-κB pathway	H22; C57BL/6 mice	RIGI induces M1 macrophages and promotes the apoptosis and death of hepatocellular carcinoma cells in vitro and in vivo without affecting normal liver cells, and inhibits the growth of hepatocellular carcinoma.	[88]
		UpregulateSIRT1	Upregulate NF-κB pathway	HepG2, RAW 264.7, HL-60 macrophages	SIRT1 overexpression enhances M1-like macrophage infiltration in HCC while inhibiting HCC cell growth, invasion, and migration.	[89]
	(M0 to M1)and(M2 to M1)	Listeria-based HCC vaccine, Lmdd-MPFG combined with PD-1 blockade; rapamycin	Upregulate NF-κB pathway through the TLR2 and MyD88 pathway	Hepa1–6/MPFG tumor-bearing mice	Lmdd-MPFG induces an increase in the number of T cells in HCC TME and promotes the production of cytokines, such as IFNγ.	[63]
		Compound Kushen injection(CKI) in combination with low-dose sorafenib	Triggering tumor necrosis factor receptor superfamily member 1 (TNFR1)-mediated NF-κB and p38 MAPK signaling cascades	C57BL/6 mice and BALB/c athymic nude mice; Hepa1-6 or LPC-H12 cells	CKI activates a pro-inflammatory response in HCC microenvironment, reduces the immunosuppression of tumor-associated macrophages, promotes the proliferation and cytotoxic activity of CD8 + t cells, and leads to apoptosis of HCC cells.	[98]
		A nanoliposome-loaded C6-ceremide (LipC6) injection	Downregulate ROS signaling	Male C57BL/6 mice; B6/WT-19 cells	LipC6 induced TAMs to differentiate into M1 phenotype, which decreased immunosuppression and increased the activity of CD8 + T cells. LipC6 slowed down tumor growth by reducing tumor cell proliferation and AKT phosphorylation and increasing tumor cell apoptosis.	[90]
	Increase the proportion of M1	Matricellular protein SPON2	Upregulate SPON2 expression	HuH7、Hep3B、SMMC-7721 (TCHu13)、MHCC-LM3、MHCC-97L 、 MHCC-97H	SPON2-α4β1 integrin signal activates RhoA and Rac1, increases F-actin recombination, and promotesM1-like macrophage recruitment, which not only promotes M1-like macrophage infiltration but also inhibits hepatocellular carcinoma metastasis.	[59]
Inhibiting tumor-promoting function		TREM‐1(triggering receptor expressed on myeloid cells-1) Inhibitor GF9	Inhibition of TREM-1 / ERK/NF-κβ / CCL20/CCR6 axis	Hepa1-6	Blocking TREM-1 pathway reduced the recruitment of CCR6 + Foxp3+ Treg improved the therapeutic effect of PD-L1 blockade eliminated the depletion of CD8 + T cells and significantly inhibited tumor progression.	[93]
		TOGA (a novel conjugate combining 18β-glycyrrhetinic acid)	Inhibition of IL-1R1/Iκβ/IKK/NF-κβ signal pathway	HepG2 and H22, xenograft nude mouse model(BALB/c nude mice) and orthotopic mouse model(Kunming mice)	TOGA inhibits IL-1R1 and then inhibits EMT mediated by IL-1 β (ligand of IL-1R1) secreted by TAMs to achieve the anti-tumor effect.	[95]

Based on the consensus that “TAMs can generally promote tumors”, several kinds of preclinical or clinical experiments have been proposed and applied: depletion of TAMs (inhibition of the monocyte/macrophage recruitment), regulation of TAM polarization, and inhibition of TAM tumor-promoting functions (targeting the molecules with tumor-promoting functions). Regardless of the progress made, these strategies have displayed some major limitations. For example, as CSF1R inhibitors were unable to specifically consume TAMs and generally can target monocytes/macrophages, but they might also damage the body’s immune system over time [86]. The use of CCR2 antagonists alone cannot completely deplete TAMs in HCC because the tissue-resident macrophages were compensatively replenished [30]. For example, simply repolarizing M2 TAMs into M1 TAMs might not be as good strategy as once thought because M1 TAMs can also play a tumor-promoting role by aiding HCC escape [57, 87]. Fortunately, recent research has begun to supplement these deficiencies. For example, promoting combined application of Notch signaling and suppressing CCL2-CCR2 signaling can completely deplete TAMs in HCC. The combination of TAM M1 polarization and PD-L1/PD-1 checkpoint blockade can render M1 TAMs “politically firm” and completely kill the liver cancer cells [57, 88]. Both the limitations of previous targeting strategies and the improvement of treatment strategies suggest that relevant treatment modalities targeting TAMs should not be rigidly confined to one specific level. The TME of HCC is complex, dynamic, and heterogeneous. Thus, potential interactions between several types of cells and components in the TME of HCC is also complicated, providing a huge network to aid HCC survival and form an almost indestructible barrier. Therefore, novel treatment strategies for HCC need to build an equal or even larger treatment network.

## Immunotherapy

**Fig. 2:**
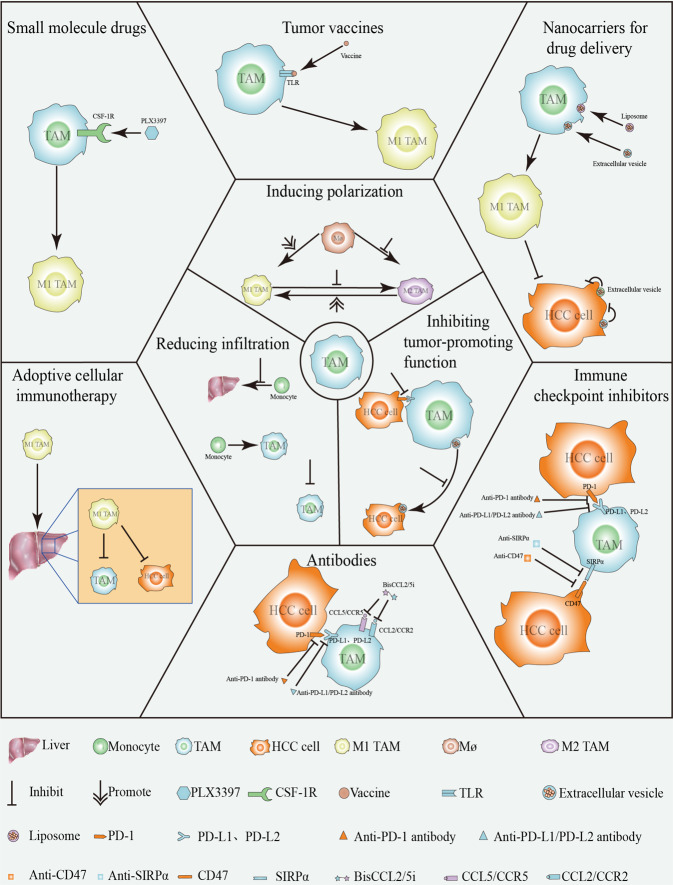
Reducing infiltration, inducing polarization, and inhibiting the tumor-promoting function have emerged as the main strategies for targeting TAMs in HCC. Thus combination of any two or more approaches between small molecular drugs, immune checkpoint inhibitors, antibodies, tumor vaccines, adoptive cellular immunotherapy, nanocarriers for drug delivery (including but not limited to) might be able to achieve effective tumor inhibition or even tumor elimination.

Macrophages play a key role in regulating the actions of tumor chemotherapy, radiotherapy, antiangiogenic drugs, and hormone therapy [89]. Their effects are complex and dual, acting as amplifiers or inhibitors of anti-tumor activity. Although considerable progress has been made in dissecting the yin and yang of macrophages in traditional anti-tumor therapy, still a major challenge remains to translate deeper knowledge into more effective treatment. Due to the increasingly prominent limitations of the traditional cancer treatment methods, a variety of new cancer treatment drugs based on immunotherapy have emerged in recent years, including small molecular inhibitors, immune checkpoint inhibitors, antibodies, tumor vaccines, adoptive cellular immunotherapy, and nanocarriers for drug delivery. Although most of these drugs do not directly or initially target macrophages, but actions of macrophages contribute to the final treatment outcome (Fig. 2).

### Small molecule drugs

Several small molecule drugs with the advantages of oral bioavailability, relatively low cost, ease of crossing physiological barriers, and entering intracellular targets have been used to target TAMs to achieve significant tumor inhibition [90]. For example, as a small molecule inhibitor, PLX3397, which is a highly specific competitive inhibitor of a CSF-1R tyrosine kinase, can change the polarization of TAMs from M2 to M1 [41]. RIPK3 (receptor-interacting protein kinase-3) deficiency in TAMs of HCC increased fatty acid oxidation (FAO) via the ROS-caspase1-PPAR (peroxisome proliferator-activated receptor) pathway, thus playing an essential role in accumulation and M2 polarization of TAMs in the TME and accelerating HCC growth [56]. Etomoxir (CPT1a inhibitor) and GW9662 (PPAR inhibitor), which are both small molecule drugs can effectively reverse accumulation of TAMs in HCC tissues, which was found to dramatically decrease Arg1 and increases iNOS in RIPK3 KO TAMs, and ablation of FAO can switch RIPK3 KO TAM polarization from M2 to M1 [56]. In addition, there is enormous potential for small molecule inhibitors to be used in combination with other tumor treatment strategies. A CCR5 antagonist (maraviroc [MVC]) was able to convert the phenotype of macrophages cocultured with irradiated liver cancer cells to M1, thereby enhancing their radiosensitivity and apoptosis [91].

### Immune checkpoint inhibitors

A number of immune checkpoint blockade therapies have been gradually identified, but the most commonly used clinical treatments are anti-PD-1 and anti-PD-L1 therapy. Overall, tumor immunotherapy based on immunosuppressive checkpoints can significantly enhance the immune response while relieving immunosuppression. Blocking the PD-1/PD-L1 pathway with pharmacological inhibitors can enhance T-cell activity and cytotoxicity, which can significantly inhibit the tumor growth of HCC [92], but the therapeutic effect is still not optimistic. Both PD-L1 and PD-1 are expressed in TAMs [93, 94]. It is worth indicating that expression of PD-L1 on M1-like TAMs can lead to immune escape of HCC [87]; thus, the combination of PD-L1/PD-1 checkpoint blockade and M1 macrophage polarization therapy appears to be a promising and effective treatment strategy [57]. Kohei Shigeta et al. treated mice with established HCC (5–6 mm in diameter) with the anti-VEGFR-2 antibody DC101 at two different doses (AA-low, 10 mg/kg and standard AA, 40 mg/kg, both thrice a week), the anti-PD-1 antibody (ICB), or their combination [88]. Anti-PD-1 immune checkpoint blockade was found to not only attenuate immunosuppression but also induced M1 macrophage polarization [88], which further enhanced the antitumor effects. In addition to PD-1/PD-L1, CD47 has been reported to act as a checkpoint associated with macrophages as a poor prognostic factor for HCC [95]. The interaction of CD47 and SIRPα on macrophages can aid the tumor cells to escape the phagocytic clearance of macrophages, though blocking CD47 can reverse macrophage-mediated tumor inhibition [96–98]. Of course, macrophage-related checkpoints are not limited to the above, but the mechanisms of other related checkpoints in HCC needs to be further explored and revealed. Fortunately, inhibition of these immune checkpoints has been found to significantly improve the effectiveness of cancer immunotherapy.

### Antibodies

The FDA has approved a number of monoclonal antibodies for the clinical management of cancers, including rituximab (for B-cell lymphoma) [99], trastuzumab (for breast cancer) [100], and immune checkpoint inhibitors. In HCC, bavituximab, a chimeric monoclonal antibody combined with sorafenib, has been reported to significantly reduce tumor microvessel density and M2 macrophage levels and increase the tumor endothelial cell apoptosis index and M1 macrophage frequency [101]. In the early stage of monoclonal antibody therapy, both the recruitment and infiltration of substantial number of M1 TAMs was considered as a sign of good prognosis [102].

In addition, known for their small size and their ease of penetrating tissue or blocking cell/protein function by binding to epitopes, single-domain antibodies might represent useful diagnostic and/or therapeutic tools that can be used as modules for various forms of antibody-based therapeutic molecules [103]. In a recent study, a bispecific single-domain antibody (BisCCL2/5i) was used in the treatment of malignant liver tumors, which could efficiently and specifically bind and neutralize CCL2 as well as CCL5, significantly induce the polarization of TAMs to the antitumor M1 phenotype, and reduce immunosuppression in the TME [104]. In addition, the combination of bispecific single domain antibody (BisCCL2/5i) and monoclonal antibody (PD-L1 inhibitor) enabled the mice to survive for a long time in liver metastasis models of the primary liver cancer, colorectal cancer, and pancreatic cancer [104].

### Tumor vaccines

As classic preventive vaccines, the hepatitis B and hepatitis C vaccines can mainly induce specific adaptive immunity before the occurrence of hepatitis by activating humoral immunity to reduce the incidence of virus-induced cancer. Although effective preventive vaccination can result in eliciting adaptive immune response, impact of immediate innate immunity on the process of spontaneous cancer regression cannot be ignored [105]. In innate immunity, dendritic cells [106, 107] and macrophages [108] can activate T cells through antigen-presenting cells to achieve the therapeutic effect of the vaccine. For example, a whole-cell GM-CSF (granulocyte-macrophage colony-stimulating factor, also called CSF-2) vaccine in combination with low-dose cyclophosphamide was reported to enhance the antigen-presenting function of the dendritic cells, neutralize the immune regulation of inhibitory Tregs (regulatory T cells), and promote activation of tumor-specific CD8 + T cells [109]. It is worth noting that GM-CSF can also induce M1 polarization of macrophages, thus enabling tumor-associated macrophages to exert their antitumor effects [110]. A *Listeria*-based HCC vaccine could activate the NF-κB pathway in TAMs through modulating the TLR2 (Toll-like receptor 2) and MYD88 (myeloid differentiation primary response protein 88) pathways, recruit p62 to activate autophagy pathways, skew M2-polarized TAMs to M1-polarized TAMs and promote PD-L1 expression in HCC cells but cause resensitization of the local tumor T cells to PD-1 immunotherapy [63]. It is worth noting that as a rising star of gene therapy, mRNA vaccines have been also applied in malignant liver tumors [104], but their safety and stability still need to be further explored.

### Adoptive cellular immunotherapy

Adoptive therapy of immune cells, which is considered as a promising method for the treatment of cancer, can induce the tumor progression inhibition or even tumor regression by transferring the specific immune cells from the host or from other donors to the tumor-bearing host. The method has been employed for the treatment of the various malignant diseases is adoptive transfer of T cells with engineered chimeric antigen receptor (CAR-Ts) [111] or genetically modified T-cell receptor (TCR-Ts) [112]. It is possible that evolutionarily conserved natural killer T (NKT) cells might be used as adoptive cell subsets to clear TAMs and liver cancer cells [113]. Nevertheless, the progress of adoptive T-cell therapy for solid tumors is relatively slow [114], which might be limited by the fact that T cells are unable fully infiltrate solid tumors such as HCC. Interestingly, M1-polarized TAMs can increase T-cell recruitment into HCC, activate T-cell cytotoxicity, and promote T-cell proliferation [82, 115]. Moreover, M1-polarized TAMs can enhance CAR T-cell activity by producing IL-12 [116]. The first generation of chimeric antigen receptors, which combine the scFv (single chain antibody fragment) of anti-CD19, anti-mesothelin, or anti-HER2 antibodies with a CD3 intracellular domain, was designed by Klichinsky et al., with further alterations as modified macrophages with the chimeric antigen receptor (CAR-Ms) for testing. These macrophages (M1-like) showed strong tumoricidal effects in various preclinical models, and even CAR-Ms maintained their antitumor activity in the presence of human M2 macrophages [117]. Although this study was not based conducted in liver tumors, it provides a practical basis for the application of macrophage adoptive therapy in HCC, which is also a solid tumor.

### Nanocarriers for the drug delivery

Due to the rapid metabolism of some drugs, excretion from the body, or non-uniform distribution in the body to weaken efficacy, drug nanocarriers (including polymer nanoparticles, liposomes, micelles, dendrimers, and inorganic nanoparticles) that can effectively target diseased areas are needed [118]. For example, MC3 LNPs (Dlin-MC3-DMA-based lipid nanoparticles) based on the liver homing deliver mRNA encoding BisCCL2/5i to the malignant liver tumors, can reduce M2 TAM infiltration and increase the proportion of M1 TAMs, and immunotherapy-related adverse events (irAEs) associated with the common complications of the systemic administration of immunotherapy are significantly decreased [104]. In the HCC model, immunosuppressive macrophages in the liver are the main types of cells that can ingest nanoparticles (NPs) [119, 120]. Thus, by reducing hypoxia-induced M2 TAM hepatocellular carcinoma infiltration, promoting macrophage polarization to M1 TAMs, increasing the number of CD8 + cytotoxic T cells in HCC, and reprogramming the immunosuppressive TME, MnO2 NPs can effectively contribute to the therapeutic strategy for PD-1/PD-L1 immune checkpoints and the effect of whole-cell tumor vaccine immunotherapy [82]. Certainly, studies based on exosomes can provide a feasible basis for constructing suitable liposomes to specifically target TAMs in HCC. For example, studies of the premetastatic niche of tumors have revealed selective uptake of exosomes by the liver resident macrophages [121, 122], thereby suggesting that it is possible to construct exosomes, liposomes, and exosome-liposome hybrid nanoparticles to target TAMs. However, nanocarriers are not used alone, but they need to be combined with other drugs to achieve regulation of TAMs in HCC to exert a substantial antitumor effects. Furthermore, as with other targeted therapies for cancer, the identification of patient populations that are likely to benefit from macrophage-targeted therapies will be essential for improving cancer treatment strategies as well as the clinical outcomes.

## Future perspectives and conclusion

TAMs have emerged as an interesting candidate for innovative anti-tumor therapy, and several new treatments have been tested to reduce the population of TAMs in the tumors. However, so far, the effect is rather limited. Recently, reprogramming M2-like TAMs into immunostimulatory and anti-tumor M1-like cells has become an attractive strategy for cancer treatment, with encouraging preclinical and preliminary clinical data. For example, some clinical trials based on the solid tumors have revealed efficacy of the targeted therapy for TAMs (including NCT02829723,NCT03447314,NCT03007732,NCT02216409, etc.). However, while tumors display the characteristics of convergent evolution, there are also some uniqueness and some differences among the solid tumors, especially with respect to TAMs populations. For instance, in the lung cancer model, bone marrow-derived macrophages promote the spread of the metastatic tumors, whereas the tissue resident macrophages support the proliferation of the cancer cells at the primary tumor site [123]. In murine ovarian cancer, a population of self-renewing CD163^+^Tim4^+^TRMs in the omentum can promote the metastatic spread by generating a protecting niche for cancer stem cells [124]. In the murine glioma, TAMs are mostly derived from the resident microglia, which can promote murine glioblastoma through regulating mTOR-mediated immunosuppression [125, 126]. In the breast cancer mouse model, the number of macrophages in the tissue decreased over time, but the number of TAMs produced by the monocytes derived from bone marrow increased [127]. In this case, the ablation of tissue resident macrophages was not found to affect the growth of the tumor, but the ablation of the circulating monocytes led to the decrease of tumor size [127]. In contrast, in the mouse model of pancreatic cancer, TRMs were amplified during the tumor development and obtained a transcriptional spectrum of a typical fibrogenic program beneficial to the pancreatic cancer, which was not destroyed by the depletion of BM-derived macrophages, but was reversed by the depletion of TRMs [128]. Interestingly, in HCC model, when the tumor formed, the resident macrophages in the liver were depleted, and some of the bone marrow-derived macrophages were effectively transformed into KCs-like TAMs after entering the original liver resident macrophage niche [24, 77]. Moreover, the simple depletion of BM-derived macrophages or liver resident macrophages was not able to achieve TAMs clearance in HCC [24, 30]. The differences among TAMs function in different tumors are primarily caused by the differences of various sources, different proportions, and various locations. Therefore, future studies related to development of the targeted therapy targeting TAMs should focus on a certain kind of tumor, such as HCC.

At present, there are only few clinical trials in HCC based on targeting TAMs, and the efficacy of these modalities need to be further evaluated (Table 3), which could be affected by the therapeutic limitations caused by high heterogeneity of macrophages. In recent years, gradually mature and perfect techniques such as single cell sequencing, metabonomic and digital spatial mapping can reveal multiple targets that can be used to specifically target TAMs from the perspectives of their functional phenotype and corresponding markers, metabolic capacity, and spatial distribution. At present, clinical trials of TAMs imaging and targeted therapy based on biological agents and macrophage markers have begun. Although these studies do not target HCC, they might provide some ideas for future targeted therapy against TAMs present in HCC. For instance, a phase I/IIa clinical trial has been launched to evaluate both the tolerance and safety of Ga [68]-labeled anti-MMR(CD206) Nb(nanobody) for PET/CT scans in cancer patients with melanoma, breast cancer, and head and neck cancer (NCT04168528). In addition, in a phase II clinical trial, inoperable solid cancer patients will undergo 68^GA^-anti-MMR Nb imaging before and after treatment, including ICIS (NCT04758650), to evaluate the prognostic value of MMR imaging. One of the key problems that need to be solved is the specificity of the targeted markers, which should be as specific as possible for identifying TAMs. Unfortunately, although markers such as CD206 and SIRPα can be used for treatment, they are not specific towards TAMs and peripheral toxicities are inevitable [129, 130].

**Table 3:** Clinical trials targeting TAMs for HCC treatment.

Agent	Target	Clinical trial number	Mechanism	Efficacy or Phase	Limitations
BMS-813160	CCR2/5	NCT04123379	Cut CCL2-CCR2 and CCL5-CCR5 axis, reduce macrophage recruitment.	Phase 2	The severe depletion of monocytes caused by inhibition of the CCL2-CCR2 signal, which leads to compensatory proliferation of resident macrophages (KC-like TAMs) in tissue to supplement the number of TAMs in the HCC microenvironment.
Regorafenib	CSF1R,VEGFR2	NCT04170556	Inhibition of CSF1R and VEGFR2, Reduce the survival of macrophages and inhibit the angiogenic effect of macrophages.	Phase 1	Due to the lack of specificity and targeting, and the depletion of macrophages, patients are prone to systemic toxicity and infection.
Chiauranib	CSF1R,VEGFR2	NCT03245190	Inhibition of CSF1R and VEGFR2, Reduce the survival of macrophages and inhibit the angiogenic effect of macrophages.	Phase 1/2	Due to the lack of specificity and targeting, and the depletion of macrophages, patients are prone to systemic toxicity and infection.
RO7119929	TLR7	NCT04338685	TLR7 agonists achieve tumor regression by inducing macrophage repolarization.	Phase 1	Although TLR agonists stimulate potent proinflammatory polarization in myeloid cells, their effects can be limited, as they induce macrophage expression of PD-L1, resulting in an inflammation-dampening effect.

A basic understanding of macrophages has led to unfolding of novel ideas for potential application of adoptive engineered macrophages, which exhibit limited phenotypes or enhanced therapeutic functions. On the other hand, macrophages display natural tumor site homing characteristics in response to the cytokines / chemokines released from TME [131]. This enables macrophages to emerge as potential therapeutic agents or diagnostic reagents to transfer to the tumor site. Therefore, three different kinds of genetic engineering therapy based on macrophages have been gradually developed: genetically engineered macrophages with enhanced therapeutic effects, macrophages as delivery tools, and macrophage derivatives as therapeutic carriers [117, 132–134].Although adoptive cell therapy using engineered macrophages has shown promising potential for the clinical application, however, mass production with strict quality control and potential biosafety problems are still two major challenges that must be overcome in the transition to the clinical application in the future. In addition, in-depth study of macrophage-mediated pharmacokinetics, pharmacodynamics, and phenotypic transformation could be of great significance to markedly improve the therapeutic efficacy and reduce side effects.

In the future, the treatment of tumor will not be limited to single or few drugs, and the combined use of multi-drugs and multi-pathways will be the inevitable trend for tumor therapy. Of course, there are still many complex problems to be solved in the dimension of TAMs. It is not clear whether the depletion of TAMs can be limited to the tumor without affecting monocytes/macrophages in the normal tissues and blood circulation? In addition, macrophage transcription factors that play a key role in promoting tumor immunosuppression and immune activation need to be identified? Moreover, it is not known whether the long-term maintenance of anti-tumor phenotype of macrophages can be regulated. Furthermore, it needs to be examined if CAR-M can infiltrate the tumors well through depletion of TAMs. In addition, although the traditional “M1/M2” dichotomy is still used in this paper, future studies should give sufficient functional and phenotypic markers on the basis of this classification, such as F4/80^+^CD11b^lo^Ly6c^lo/−^TAMs, which should be able to more clearly reveal the different subsets of TAMs present in TME and pave the way for the future development of multi-targeted drugs.

## Data Availability

All data generated or analyzed in this study are included in this published article.
